# Constant-Moderate and High-Intensity Interval Training Have Differential Benefits on Insulin Sensitive Tissues in High-Fat Fed Mice

**DOI:** 10.3389/fphys.2019.00459

**Published:** 2019-04-25

**Authors:** Sergio F. Martinez-Huenchullan, Linda A. Ban, Luisa F. Olaya-Agudo, Babu Raja Maharjan, Paul F. Williams, Charmaine S. Tam, Susan V. Mclennan, Stephen M. Twigg

**Affiliations:** ^1^Greg Brown Diabetes & Endocrinology Research Laboratory, Charles Perkins Centre, Faculty of Medicine and Health, University of Sydney, Sydney, NSW, Australia; ^2^School of Physical Therapy, Faculty of Medicine, Universidad Austral de Chile, Valdivia, Chile; ^3^Department of Biochemistry, School of Medicine, Patan Academy of Health Sciences, Lalitpur, Nepal; ^4^Northern Clinical School and Centre for Translational Data Science, University of Sydney, Sydney, NSW, Australia; ^5^NSW Health Pathology, Sydney, NSW, Australia; ^6^Department of Endocrinology, Royal Prince Alfred Hospital, Sydney, NSW, Australia

**Keywords:** high-fat diet, obesity, insulin resistance, exercise, skeletal muscle

## Abstract

In a mouse model of diet-induced obesity, this study determined if two exercise prescriptions with equivalent time and distance covered, [constant-moderate endurance (END) and high intensity interval training (HIIT)], exert differential metabolic benefits on insulin sensitive tissues. Male 10 week old C57BL/6 mice were fed a high fat diet (HFD; 45% kcal fat) *ad libitum* for 10 weeks and for a further 10 weeks they underwent END or HIIT training (3 × 40 min sessions/wk). Untrained HFD and chow-fed mice acted as controls. At 30 weeks of age, mice were sacrificed and *quadriceps* muscle, subcutaneous adipose tissue (SAT) and liver were excised. Neither END nor HIIT altered body weight or composition in HFD mice. In *quadriceps*, HFD decreased high-molecular weight adiponectin protein, which was normalized by END and HIIT. In contrast, HIIT but not END reversed the HFD-driven decrease in the adiponectin receptor 1 (AdipoR1). In SAT, both programs tended to decrease collagen VI protein (*p* = 0.07–0.08) in HFD, whereas only HIIT induced an increase in the mRNA (3-fold vs. HFD untrained) and protein (2-fold vs. HFD untrained) of UCP1. In liver, only END reversed collagen I accumulation seen in HFD untrained mice. Our results suggest that HIIT may promote better systemic metabolic changes, compared to END, which may be the result of the normalization of muscle AdipoR1 and increased UCP1 seen in SAT. However, END was more effective in normalizing liver changes, suggesting differential metabolic effects of END and HIIT in different tissues during obesity.

## Introduction

Obesity is a pervasive metabolic disorder, with complications ranging from insulin resistance and type 2 diabetes, to cardiovascular disease, and hepatic lipid accumulation that can lead to fibrosis. Given the metabolic benefits that it can confer even in the absence of changes in body weight and/or composition (Winn et al., 2018), physical exercise is one of the most frequently prescribed lifestyle modifications used to manage these conditions. The type, intensity, and duration of exercise are all known to have an effect on clinical outcomes (ACSM, 2016), which may be due to the metabolic adaptations seen in insulin sensitive tissues, such as skeletal muscle, white adipose tissue and liver (Cho et al., 2015; Chavanelle et al., 2017; Wang et al., 2017). Bajpeyi et al. (2009) were one of the first to report that longer exercise sessions conferred improvement in whole-body insulin sensitivity in overweight or obese people during and after 8 months of exercise. These results also highlighted the relevance of controlling for session length when comparing different training programs. In the context of normalizing metabolic disturbances, aerobic exercises such as constant-moderate endurance training (END) and high-intensity interval training (HIIT) are popular amongst health care professionals. This is due to the ability of aerobic activity to improve systemic parameters such as aerobic performance (Milanovic et al., 2015), body composition (Keating et al., 2017; Wewege et al., 2017), vascular function (Ramos et al., 2015), and glucose regulation (Jelleyman et al., 2015). However, studies which aim to elucidate the molecular mechanisms behind these effects are few and have been incompletely documented. Chavanelle et al. (2017) in *db/db* mice compared effects of 10 weeks of HIIT and END on insulin signaling and mitochondrial function in the *gastrocnemius* muscle. They found that HIIT but not END improved whole-body insulin sensitivity and increased the GLUT4 muscle protein content, but did not produce major changes in mitochondrial function or insulin signaling parameters (i.e., pAMPK/AMPK) (Chavanelle et al., 2017). END and HIIT programs have caused similar changes in insulin sensitivity in livers of high-fat fed mice. HIIT, had a greater protective effect in terms of restoring hepatic adiponectin receptor 2 levels, pAMPK/AMPK ratio, and down-regulation of NF-κB (Cho et al., 2015). In the subcutaneous adipose tissue of these mice, HIIT promoted improvements in insulin signaling (i.e., pAkt/Akt ratio) and increased the protein level of mitochondrial biomarkers (Wang et al., 2017).

Despite the described benefits of these previous studies in obese animals which favored HIIT over END in metabolic benefits, there were limitations in study design. Some of these studies included low aerobic intensity in the END protocol (Cho et al., 2015), major differences in the training intensities between programs that relied upon distance covered rather than duration of exercise (Wang et al., 2017), and the inclusion of treadmill inclinations without considering the confounding effect of body weight (Linden et al., 2015, 2016; Wang et al., 2017). Together these differences make it difficult to make head to head comparisons between the END and HIIT exercise programs. Indeed, these caveats were emphasized in a study, albeit for a shorter duration of 4 weeks of energy-matched HIIT and END, there was no differential benefit between the exercise programs in terms of decreased intrahepatic fat, lower postprandial circulating insulin, c-peptide secretion and lipid peroxidation levels in overweight or obese people (Winn et al., 2018). As this study was performed in humans, the effect on metabolic indicators was not examined in insulin sensitive tissues. Therefore, it remains to be determined whether these described benefits of HIIT compared to END on insulin sensitive tissues would be present if the training programs were equivalent in terms of distance traveled, time per session, and average intensity.

In the light of the aforementioned findings, the purpose of this study was to compare the effect of HIIT and END programs on the metabolic function of skeletal muscle, white adipose tissue, and liver in obese mice. These two exercise regimens were designed to be equivalent in terms of distance covered, time per session, and average intensity of exercise. We hypothesized that both programs would confer similar metabolic benefits on the three insulin sensitive tissues of interest.

## Materials and Methods

### Ethics Statement

This study was approved by the University of Sydney Animal Ethics Committee (Protocol #2015/816). The experiments described herein were carried out according to the guidelines laid down by the New South Wales Animal Research Act and the 8th Edition of the Australian code for the care and use of animals for scientific purposes.

### Animal Characteristics

Sample size for this study was calculated as follows: considering previous studies from our group (Martinez-Huenchullan et al., 2018) and that one of our main outcomes was muscle high-molecular weight (HMW) adiponectin, having a statistical significance of <0.05; power of 0.8 and assuming a variance of 25% in the outcome of interest, the minimum number of mice per group should be 8 to 10. We added extra animals to account for lack of exercising in some mice (reportedly about 10–20%). Therefore, seventy-two male mice of 10-weeks of age were used in this study (Animal Resources Centre, Perth, Australia). Animals were randomly distributed into two dietary groups (*n* = 36/group) and fed either an in-house high-fat diet (HFD; 45% kcal fat) prepared as per Lo et al. (2011), or standard laboratory chow (CHOW; 12% kcal fat) (Meat free mouse diet, Specialty feeds^®^, WA, Australia), *ad libitum* for 10 weeks. After 10 weeks, each of the dietary groups (HFD or CHOW) were randomized to either no additional exercise, a constant moderate-intensity endurance (END) exercise program or a HIIT exercise program. Therefore, this study had six experimental groups (*n* = 12/group): CHOW no exercise (untrained), CHOW+END, CHOW+HIIT; and HFD untrained, HFD+END, HFD+HIIT. Dietary conditions and exercise interventions were continued simultaneously from weeks 11 to 20, and phenotyping studies were performed in the following week after exercise intervention. Animals were euthanized under isoflurane anesthesia by exsanguination through cardiac puncture, blood, *quadriceps* muscle, subcutaneous adipose tissue, and liver were collected and stored appropriately for later analysis. Samples were collected at least 72 h after the last exercise session.

### Exercise Training

All animals underwent 1 week of treadmill acclimation (6 m/min for 10 min). Afterward, a maximal running capacity (MRC) test was performed. This test started at 6 m/min and progressively the speed was increased 3 m/min every 3 min until exhaustion. This was defined as the inability of the mouse to reach the end of the treadmill lane after 5 mechanical stimuli (soft brush) were delivered within 1 min. The MRC (100%) was defined as the maximal speed reached during the test, and the distance covered was considered the aerobic performance of the animal. The exercise intensity of the two different training programs was matched using the initial MRC. The exercise programs were as follows: END was defined as a running session at 70% of the MRC (CHOW = 19 m/min; HFD = 15 m/min) for 40 min, while HIIT consisted in eight bouts (2.5 min each) at 90% of the MRC (CHOW = 24 m/min; HFD = 20 m/min). Between each bout, active rest periods (2.5 min) at 50% of the MRC (CHOW = 14 m/min; HFD = 10 m/min; 40 min total per session) were performed. Given that the intra-group MRC variability between animals was very low at less than 10%, the realized exercise prescription was the same in all mice within the same experimental group. The two exercise regimens were considered to have established equivalence between END and HIIT in terms of running distance covered, session timing, and average exercise intensity. Each session was performed during the morning, three times per week, for 10 weeks. Untrained animals only performed running activity during the acclimation period and the performance of the MRC test. The MRC test was subsequently reapplied at the end of the training programs.

### Animal Phenotyping

Body weight (g) was assessed once per week. Body composition and systemic insulin sensitivity measurements were performed 72 h after the last exercise session. Fat and lean mass were measured by EchoMRI (EchoMRI^TM^ 900 system, Houston, TX, United States). Insulin sensitivity was measured by insulin tolerance test (ITT) (Ayala et al., 2010) as previously described (Lo et al., 2011). Briefly, after 4 h of fasting, baseline tail vein blood glucose level was measured (approx. 2 μl) using a standard glucometer (FreeStyle Lite, Abbott Diabetes Care, Alameda, CA, United States). Insulin (Actrapid^®^, Novo Nordisk^TM^) was administered then intraperitoneally (0.75 IU/kg of body weight). Afterward, consecutive blood glucose level measurements were conducted at 5, 15, 30, and 60 min after insulin injection from the tail vein. Blood glucose excursion was calculated as Area Under the Curve (AUC) (Lo et al., 2011).

### Muscle Function Testing

Muscle function was measured through two non-invasive tests previously validated in our laboratory (Martinez-Huenchullan et al., 2017), namely grip strength and hang wire test. To measure grip strength performance, a Chatillon DFIS 2 Force Gauge was used (Martinez-Huenchullan et al., 2017), where mice were tested three times each with a 60 s rest between trials. The mean value between the three trials was considered for statistical analysis. For the hang wire test, each mouse was placed on a suspended wire (35 cm height) over a padded area (3 min maximum). The number of times the animal reached either end of the wire was counted, same procedure in terms of the number of times the mouse fell from the wire. The test was stopped if the animal fell 10 times (van Putten, 2016). This entire test was performed once only (Martinez-Huenchullan et al., 2017). As the exercise regimes may cause aerobic adaptation another maximal running capacity test was performed at the end of the experiment (i.e., at week 20).

### Biochemical Analysis of Plasma and Muscle Tissue

Commercially available kits were used to measure plasma insulin (Merck^®^, catalog number EZRMI-13K), soluble tumor necrosis factor (TNF) (R&D Systems^®^, catalog number MTA00B), and fibroblast growth factor 21 (FGF-21; Abcam^®^, catalog number ab212160) and triglycerides (TAG; Sigma^®^, catalog number: TR0100). Alkaline phosphatase (ALP), alanine transaminase (ALT), and aspartate transaminase (AST) were determined on a Roche^®^ Cobas auto-analyzer in the Clinical Chemistry Laboratories of NSWHP, Royal Prince Alfred.

To quantify muscle TAG content, lipids were extracted from approx. 10 mg of muscle with chloroform-methanol (2:1) and they were measured using the Roche^®^ TAG reagent (catalog number 11876023). Muscle TAG content was then calculated from the standard curve and expressed as mg/mg of tissue. Considering that both exercise programs had differences in intensities, and therefore, in the muscle fibers activated, we selected a muscle that, because of their composition, would be affected by both training programs. In this scenario, *quadriceps* fitted the criteria considering that it is a mixed-fiber muscle (Yamada et al., 2012). An additional reason is that, metabolically, mouse quadriceps behaves very similarly to human vastus lateralis (Jacobs et al., 2013), and therefore the findings derived from this tissue may be more directly extrapolated to a human condition.

### RNA Extraction, Reverse Transcription, and Real Time-qPCR

Total RNA was extracted from muscle (50–60 mg), subcutaneous adipose tissue (150 mg) and liver (40–50 mg) using TRI reagent (Sigma^®^, catalog number T9424). RNA yield and quality were measured spectrophotometrically (Thermo Fisher Scientific, NanoDrop^®^, Waltham, MA, United States) and were considered acceptable if the 260/230 ratio was above 1.8. cDNA synthesis was performed using 1000 ng of RNA, oligo(dT) 0.5 μg/μL (Life Technologies, catalog number 18418-012) and Superscript III reverse transcriptase 200 U/μL (Life Technologies, catalog number 18080-093). Real time-qPCR for skeletal muscle and adipose tissue mRNAs were performed using SensiMix SYBR Green (Bioline, catalog number QT605-20) on a Rotor-gene Q thermocycler (QIAGEN^®^, Hilden, Germany). The no-template control included in each run as a negative control failed to amplify, and the melt curve analysis showed a single peak at the appropriate temperature. Liver mRNA species were determined using TaqMan probes a custom designed array (OpenArray^TM^, Thermo Fisher Scientific^®^) and were amplified using the QuantStudio^TM^ 12 K Flex real-time PCR system for analysis according to standard protocols. Results were expressed using the delta-delta Ct method, correcting values using the housekeeper genes, Rpl7L1 for skeletal muscle as previously published (Thomas et al., 2014), NONO for adipose tissue, and Aldolase B (AldoB) for liver. The list of primer sequences and TaqMan formats are provided in Supplementary Table S1.

### Protein Analysis by Western Immunoblot

Protein levels of adiponectin, adiponectin receptor 1 (AdipoR1), Collagen I, Collagen IV, connective tissue growth factor (CTGF), transforming growth factor (TGF)-beta, and GLUT4 were determined in muscle, liver, and plasma by Western immunoblot, using established methods (Tam et al., 2015). Briefly, *quadriceps* muscle (25–30 mg) or liver (10–15 mg) were homogenized in ice-cold radioimmunoprecipitation assay buffer (RIPA). Proteins were extracted and quantified using a detergent compatible (DC^TM^) protein assay (Bio-Rad^®^, DC^TM^ catalog number 500-113 and 500-114). The muscle or liver samples containing 40 μg of protein or 0.5 μL of plasma were run on polyacrylamide gradient gels (4–15%) (Bio-Rad^®^, catalog number 4568086). The proteins contained within the gel were transferred to a nitrocellulose membrane (Bio-Rad^®^, catalog number 1704158) at 120 V using the Trans-Blot^®^ turbo^TM^ Transfer System (Bio-Rad, Hercules, CA, United States). Proteins of interest were detected with primary antibodies and dilutions as detailed in the Supplementary Table S2 after overnight incubation at 4°C. Then, membranes were incubated with peroxidase labeled secondary antibodies (Anti-rabbit IgG 1:10000, Sigma^®^, catalog number S9169; Anti-mouse IgG 1:10000, Sigma^®^, catalog number A9044) for 1 h at room temperature. Membranes were then washed with tris buffered saline plus Tween 20 (TBST) and developed with a chemiluminescent substrate (Clarity^TM^ Western ECL substrate, Bio-Rad^®^, catalog number 170-5061) and visualized on a ChemiDoc imaging system (Bio-Rad^®^, Hercules, CA, United States). Densitometric analysis of the bands was performed using Image Lab software (Bio-Rad^®^, Hercules, CA, United States). Protein loading was confirmed, and specific band intensities were normalized using Ponceau S staining.

### Histology and Immunohistochemistry

Detection of adiponectin and GLUT4 in *quadriceps* muscle, and uncoupling protein 1 (UCP-1) and Collagen VI in subcutaneous adipose tissue using immunohistochemistry were also undertaken using standard methods (Lo et al., 2011). Briefly, samples after fixation in 10% formalin for 24 h underwent overnight processing at the Histopathology Laboratory at the Charles Perkins Centre, University of Sydney. From paraffin-embedded blocks, 5 μm sections were stained with hematoxylin and eosin to investigate the impact of HFD and the exercise on muscle and liver structure. Also, in 5 μm sections, immunohistochemistry was used to examine the intensity and location of Adiponectin (skeletal muscle), GLUT4 (skeletal muscle), UCP-1 (adipose tissue), and Collagen VI (adipose tissue). After antigen retrieval (microwave at pH 6.0 citrate buffer for 30 min), the slides were incubated overnight at 4°C with primary antibodies as detailed in Supplementary Table S2. After secondary antibody incubation for 30 min (1:200, Biotinylated anti-rabbit IgG, Vector Laboratories^®^, catalog number BA-1000) at room temperature, color was developed using avidin-biotin complex (Vectastain ABC kit, Vector Laboratories^®^, catalog number PK-4000) with diaminobenzidine (DAB) (Dako^®^, catalog number K3468). The reaction was terminated by washing the slides in ddH_2_O and the slides were imaged using an Olympus BX53 microscope, with cellSens microimaging software (Olympus, Tokyo, Japan). Quantification of percentage positive staining in each slide was achieved using the image processing software Fiji^®^ (Schindelin et al., 2012).

### Statistical Analysis

Statistical analyses were performed using GraphPad Prism software Version 7.0. Normally distributed data were expressed as mean ± SD. Non-normally distributed data were expressed as median [interquartile range]. To assess the effects of dietary interventions (CHOW vs. HFD) along with the differences between exercise regimes (untrained vs. END vs. HIIT), two-way ANOVA analysis were conducted with Tukey’s and Sidak’s *post-hoc* tests. Differences to be considered as significant, associated *p-*values had to be less than 0.05.

## Results

### Diet and Exercise Effects on Animal Characteristics

Most animals completed the exercise programs without difficulty however, three animals were withdrawn at various time points due to lack of compliance to the exercise regimen (HFD+END *n* = 1, HFD+HIIT *n* = 2). In terms of physical outcomes, animals fed HFD gained weight and this weight gain was not impacted by either END or HIIT (Table 1), where only HIIT tended to decrease this parameter (*p* > 0.05). In body composition, a lower fat to lean mass ratio was seen in CHOW mice only after exercise, indicative of reduced fat mass (Table 1). In the HFD unexercised animals, *quadriceps* weight was lower compared to similarly treated age matched controls, and this difference was not seen after both exercise programs (Table 1). After 10 weeks of training, only animals after HIIT exhibit lower amount of subcutaneous adipose tissue (*p* < 0.05), whereas in liver, the expected increases in liver weight after 20 weeks of HFD were not seen after both exercise programs (both *p* < 0.05, Table 1).

**Table 1:** Animal characteristics at termination.

Parameter	CHOW			HFD		
		
	Untrained (*n* = 12)	END (*n* = 12)	HIIT (*n* = 12)	Untrained (*n* = 11)	END (*n* = 11)	HIIT (*n* = 10)
Body weight (g)	33.3 ± 2.4	32.2 ± 1.7	32.9 ± 1.2	47.7 ± 2.6^∗^	47.5 ± 4.1^∗^	45.4 ± 4.7^∗^
Fat mass (%BW)	12.1 ± 3.3	7.8 ± 1.6^∗^	8.5 ± 2.8^∗^	38.0 ± 2.4^∗^	38.1 ± 4.2^∗^	34.6 ± 6.1^∗^
Lean mass (%BW)	82.8 ± 3.3	87.4 ± 1.5^∗^	86.8 ± 2.9^∗^	58.1 ± 2.3^∗^	58.2 ± 4.2^∗^	61.7 ± 5.9^∗^
Fat/Lean mass (ratio)	0.15 ± 0.05	0.09 ± 0.02^∗^	0.10 ± 0.04^∗^	0.66 ± 0.07^∗^	0.66 ± 0.12^∗^	0.57 ± 0.16^∗^
Quadriceps weight (g)	0.25 ± 0.02	0.24 ± 0.02	0.25 ± 0.03	0.22 ± 0.02^∗^	0.26 ± 0.03^#^	0.26 ± 0.02^#^
Subcutaneous adipose tissue (%BW)	1.05 ± 0.25	0.90 ± 0.15	0.89 ± 0.19	3.78 ± 0.46^∗^	3.55 ± 0.78^∗^	3.01 ± 0.75^∗#^
Liver weight (%BW)	4.59 ± 0.42	4.52 ± 0.32	4.35 ± 0.55	7.71 ± 0.85^∗^	6.16 ± 1.56^∗#^	5.67 ± 0.79^∗#^


*Data are expressed as mean ± SD. Percentage of body weight (%BW). ^∗^*p* < 0.05 vs. CHOW untrained; ^#^*p* < 0.05 vs. HFD untrained by Two-way ANOVA with Tukey’s and Sidak’s *post hoc* tests.*

**FIGURE 1:**
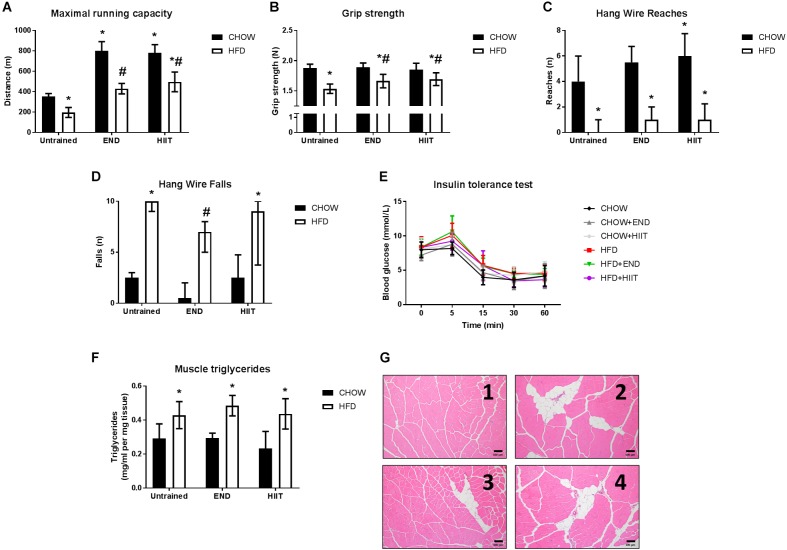
Muscle function, structure, and systemic insulin sensitivity after training. **(A)** Distance covered in the maximal running capacity test, **(B)** grip strength, **(C)**, number of reaches, **(D)** number of falls during the hang wire test, **(E)** blood excursion during the insulin tolerance test, **(F)** muscle triglycerides levels, and **(G)** representative cross-sectional quadriceps muscle sections, with haematoxylin and eosin staining at 10× magnification for CHOW untrained (1), HFD untrained (2), HFD+END (3), and HFD+HIIT (4). Data are presented as mean ± SD **(A,B,E)** and median [IQR] **(C,D)** with *n* = 6–12 animals per group. ^∗^*p* < 0.05 vs. CHOW untrained; ^#^*p* < 0.05 vs. HFD untrained by Two-way ANOVA with Tukey’s and Sidak’s *post hoc* tests. Statistical analysis for blood glucose excursion during the insulin tolerance test (Figure 1E) is described in Table 2.

**FIGURE 2:**
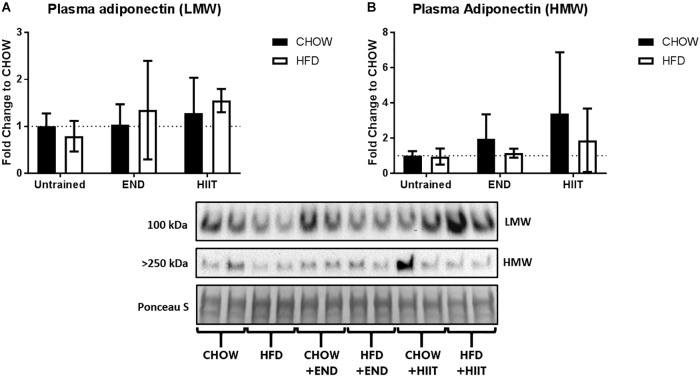
Plasma levels of adiponectin isoforms. **(A)** Low molecular weight (LMW) and **(B)** high molecular weight (HMW) adiponectin in plasma. Data are presented as mean ± SD and the number of animals per group is 8. ^∗^*p* < 0.05 vs. CHOW untrained; ^#^*p* < 0.05 vs. HFD untrained by Two-way ANOVA with Tukey’s and Sidak’s *post hoc* tests.

Neither diet nor exercise influenced fasting blood glucose, whereas high-fat fed mice exhibited higher levels of plasma insulin, which that was not affected by any of the exercise programs. Interestingly, in HFD mice HIIT but not END the higher levels of the AUC of the blood glucose excursion was not seen (Table 2 and Figure 1E). To examine liver health, circulating ALP, ALT, and AST were assessed. Compared with CHOW, HFD induced a significant change in all three parameters, which were milder after both END and HIIT (all *p* < 0.05) (Table 2). As previously described (Gao et al., 2010), high-fat fed mice showed lower plasma triglycerides levels, a change that was not present after END. No major changes were seen in plasma TNF and the expected higher levels of FGF-21 (Fisher et al., 2010) after HFD were not seen after both END and HIIT (both *p* < 0.05; Table 2). No effects of diet or exercise were seen in the circulating levels of low molecular weight (LMW) or high molecular weight (HMW) adiponectin (Figure 2 and Supplementary Figure S1).

**Table 2:** Circulating metabolic factors.

Parameter	CHOW			HFD		
		
	Untrained (*n* = 12)	END (*n* = 12)	HIIT (*n* = 12)	Untrained (*n* = 11)	END (*n* = 11)	HIIT (*n* = 10)
Fasting blood glucose (mmol/L)	7.98 ± 1.14	7.47 ± 0.85	8.1 ± 1.61	8.61 ± 1.34	8.37 ± 1.17	8.33 ± 1.42
Plasma insulin (ng/mL)	2.41 ± 3.09	1.19 ± 0.91	1.07 ± 0.61	9.08 ± 7.93^∗^	24.89 ± 10.59^∗#^	10.09 ± 5.44^∗^
Insulin tolerance test (blood glucose AUC)	275 ± 52	282 ± 64	310 ± 61	336 ± 53^∗^	336 ± 31^∗^	298 ± 64
Alkaline phosphatase (fold change from CHOW untrained)	1.00 ± 0.25	1.00 ± 0.24	0.99 ± 0.21	1.82 ± 0.87^∗^	1.18 ± 0.56^#^	1.09 ± 0.72^#^
Alanine transaminase (fold change from CHOW untrained)	1.00 ± 0.18	1.92 ± 1.01	1.20 ± 0.47	10.94 ± 6.10^∗^	5.83 ± 4.11^#^	3.93 ± 3.99^#^
Aspartate transaminase (fold change from CHOW untrained)	1.00 ± 0.22	2.07 ± 1.03	1.16 ± 0.49	3.33 ± 2.13^∗^	1.98 ± 0.71^#^	1.89 ± 1.21^#^
Plasma triglycerides (mg/mL)	1.83 ± 0.61	1.80 ± 0.57	1.33 ± 0.39	1.12 ± 0.29^∗^	1.77 ± 0.71^#^	1.44 ± 0.68
Plasma TNF (pg/mL)	1.40 ± 0.67	1.21 ± 0.67	1.35 ± 1.50	2.05 ± 1.24	1.32 ± 0.79	2.46 ± 2.20
Plasma FGF-21 (pg/mL)	504 ± 562	282 ± 282	326 ± 273	1415 ± 545^∗^	899 ± 512^#^	649 ± 496^#^


*Data are expressed as mean ± SD. Area under the curve (AUC), tumor necrosis factor (TNF), fibroblast growth factor (FGF). ^∗^*p* < 0.05 vs. CHOW untrained; ^#^*p* < 0.05 vs. HFD untrained by Two-way ANOVA with Tukey’s and Sidak’s *post hoc* tests. Samples collected for plasma insulin measurements were obtained during tissue harvest, therefore no fasting periods were considered.*

### Effects of Diet and Exercise on Skeletal Muscle

The aerobic performance determined by distance covered during the maximal running capacity test of HFD unexercised mice was lower compared to CHOW fed animals. Significant changes in this outcome were seen after both END and HIIT in high-fat fed mice (Figure 1A). High-fat fed animals showed less muscle strength, with lower grip strength performance, as a change that was attenuated after both END and HIIT (Figure 1B). In contrast, the submaximal muscle performance determined in the hang wire test (Figure 1C,D), only in HFD+END animals the number of falls was lower compared to the HFD untrained mice (Figure 1D). As expected, HFD mice exhibited higher levels of muscle triglycerides, which was unchanged by either END or HIIT (both *p* > 0.05; Figure 1F). This pattern of higher lipid content was also seen in the histological analysis of *quadriceps* muscle, where signs of fat infiltration were observed in all the HFD animals (Figure 1G).

**FIGURE 3:**
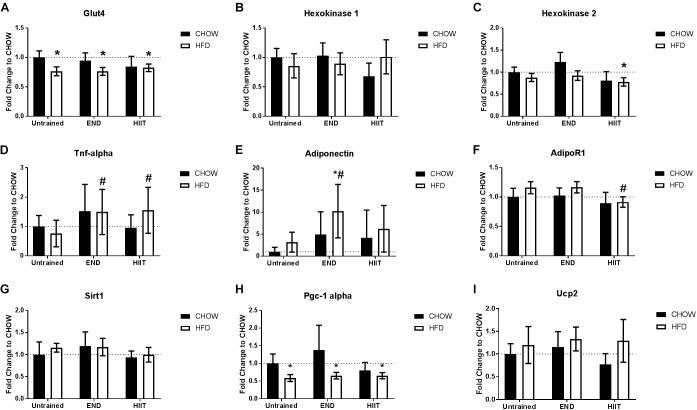
Skeletal muscle mRNA levels for glucose uptake and metabolism related genes. Glut4 receptors **(A)**, Hexokinase 1 **(B)**, and 2 **(C)**, Tnf **(D)**, muscle adiponectin **(E)**, adiponectin receptor 1 (AdipoR1; **F**), sirtuin 1 (Sirt1; **G**), peroxisome proliferator-activated receptor gamma coactivator 1-alpha (Pgc-1 alpha; **H**), and uncoupling protein 2 (Ucp2; **I**). Data are presented as mean ± SD with *n* = 10–12 animals per group. ^∗^*p* < 0.05 vs. CHOW untrained; ^#^*p* < 0.05 vs. HFD untrained by Two-way ANOVA with Tukey’s and Sidak’s *post hoc* tests.

To explore the metabolic response of skeletal muscle to HFD and exercise type, the mRNA levels of key glucose uptake and metabolism-related genes was determined. HFD mice showed lower Glut4 mRNA which was unchanged by exercise (Figure 3A). There were no major dietary or exercise effects on hexokinase 1 mRNA (Figure 3B), but after HIIT, lower levels of hexokinase 2 mRNA were seen (*p* < 0.05; Figure 3C). HFD did not have a significant effect on Tnf mRNA, however, after both exercise programs, HFD mice exhibited higher Tnf mRNA (<2-fold; Figure 3D). END training in HFD mice resulted in higher muscle adiponectin mRNA (10-fold; *p* < 0.05), that was not replicated in HFD+HIIT mice (Figure 3E). In terms of muscle adiponectin receptors (AdipoR), the mRNA levels of AdipoR1 were not affected by diet, however, after HIIT high-fat fed mice had higher levels compared to their untrained peers (*p* < 0.05; Figure 3F). The transcription levels of three genes regulated by adiponectin, were also studied. The mRNA levels of two of these: sirtuin 1 (Sirt1) and uncoupling protein 2 (Ucp2), were not altered by diet or exercise (Figure 3G–I). In contrast HFD mice showed lower peroxisome proliferator-activated receptor gamma coactivator 1-alpha (Pgc-1 alpha), a change that was unaffected by exercise (Figure 3H).

**FIGURE 4:**
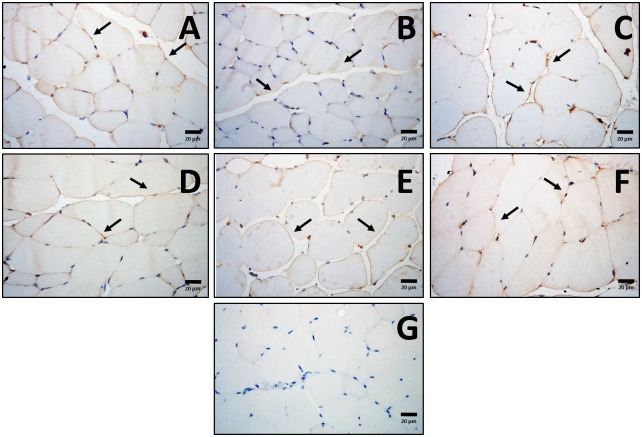
Localization of muscle adiponectin. Representative cross-sections of quadriceps muscle with adiponectin immunohistochemistry determined by immunoperoxidase stain, and nuclear counter-staining with haematoxylin. CHOW untrained **(A)**, CHOW+END **(B)**, CHOW+HIIT **(C)**, HFD untrained **(D)**, HFD+END **(E)**, and HFD+HIIT **(F)** are displayed. No striking differences were seen across the different groups. Positive staining was mainly found in the sarcolemma followed by a less intense signal in the cytoplasmic region as indicated by arrows. A negative control without primary antibody is shown in **(G)**. The size bars indicate 20 μm and images are at 40× magnification.

**FIGURE 5:**
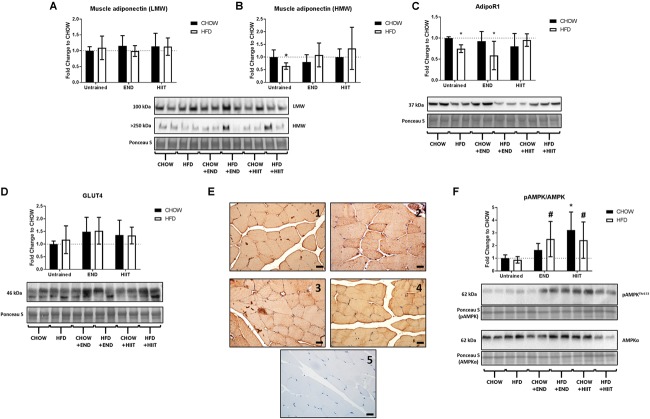
Adiponectin isoforms in muscle, adiponectin receptor 1, GLUT4, and Phospho-AMPK^Thr172^/AMPK ratio. Muscle low-molecular weight (LMW) adiponectin **(A)** and high-molecular weight (HMW) adiponectin **(B)**; muscle adiponectin receptor 1 (AdipoR1; **C**), muscle GLUT4 **(D)**, muscle Phospho-AMPK^Thr172^/AMPK ratios **(F)** and their representative blots below its respective graph with its membrane stained with Ponceau S used as loading control. Data are presented as means ± SD and the number of animals in each group is 8. ^∗^*p* < 0.05 vs. CHOW untrained; ^#^*p* < 0.05 vs. HFD untrained by Two-way ANOVA with Tukey’s and Sidak’s *post hoc* tests. Representative cross-sections of quadriceps muscle with GLUT4 immunohistochemistry determined by immunoperoxidase stain **(E)**, and nuclear counter-staining with haematoxylin. CHOW untrained (1), high-fat diet untrained (2), HFD+END (3), and HFD+HIIT (4) are displayed. A negative control without primary antibody is shown in (5). The size bars indicate 20 μm and images are at 40× magnification. As no clear differences were seen in CHOW mice before vs. after exercise training, those images are not shown.

To corroborate these results from a protein perspective, histologically in the *quadriceps* muscle, comparable expression of adiponectin, primarily localized to the sarcolemma was seen across all treatment groups (Figure 4). Western immunoblot analysis showed no major effects of diet or exercise in low-molecular weight (LMW) adiponectin (Figure 5A), however, HFD mice showed lower levels of high-molecular weight (HMW) isoform (0.6-fold; *p* < 0.05) change that was not seen after END or HIIT (Figure 5B and Supplementary Figure S2). HFD mice also exhibited lower muscle AdipoR1 (*p* < 0.05), a change that was not present after HIIT (Figure 5C and Supplementary Figure S3). In terms of GLUT4, no dietary or exercise effects were seen in total GLUT4 amount through western immunoblotting (Figure 5D and Supplementary Figure S4), or in terms of cellular GLUT4 localization detected by immunohistochemistry (Figure 5E). The ratio of Phospho-AMPK^Thr172^/AMPK was increased after exercise to a similar degree in both exercise groups (∼2-fold), whereas in CHOW-fed mice, only HIIT had a significant effect (Figure 5F and Supplementary Figures S5, S6).

### Effect of Diet and Exercise on Subcutaneous Adipose Tissue

**FIGURE 6:**
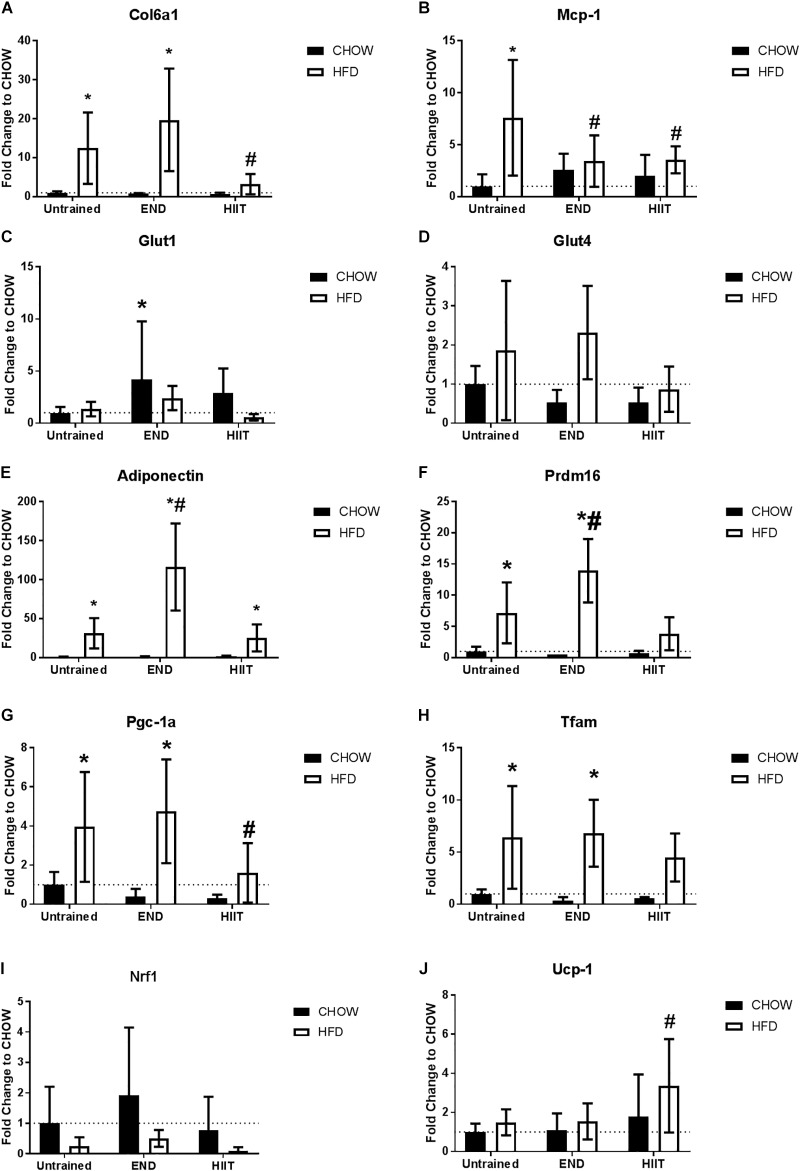
Subcutaneous adipose tissue mRNA levels of metabolism-related genes. Collagen VI alpha 1 (Col6a1; **A**), monocyte chemotactic protein-1 (Mcp-1; **B**), Glut1 receptor **(C)**, Glut4 receptor **(D)**, Adiponectin **(E)**, PR domain containing 16 (Prdm16; **F**), peroxisome proliferator-activated receptor gamma coactivator 1-alpha (Pgc-1 alpha; **G**), Mitochondrial transcription factor A (Tfam; **H**), Nuclear respiratory factor 1 (Nrf1; **I**), and uncoupling protein 1 (Ucp-1; **J**). Data are presented as means ± SD and the number of animals in each group is 6–12. ^∗^*p* < 0.05 vs. CHOW untrained; ^#^*p* < 0.05 vs. HFD untrained by Two-way ANOVA with Tukey’s and Sidak’s *post hoc* tests.

**FIGURE 7:**
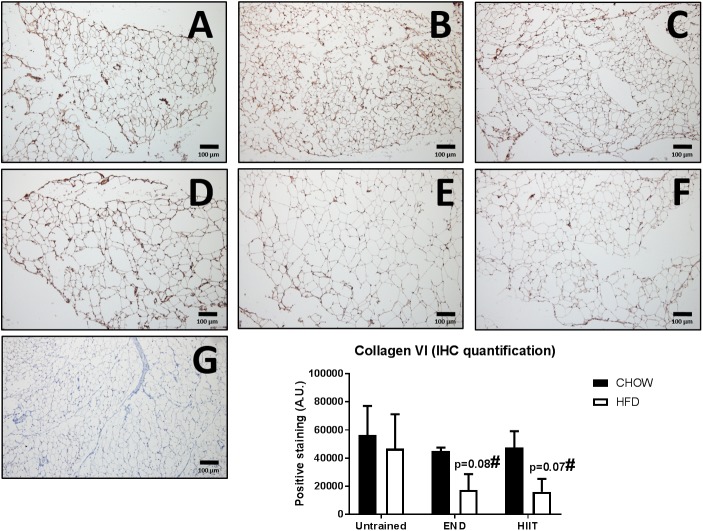
Immunohistochemistry and quantification of subcutaneous adipose tissue collagen VI. Representative cross-sections of subcutaneous adipose tissue with uncoupling protein 1 immunohistochemistry determined by immunoperoxidase stain, and nuclear counter-staining with haematoxylin. CHOW untrained **(A)**, CHOW+END **(B)**, CHOW+HIIT **(C)**, HFD untrained **(D)**, HFD+END **(E)**, and HFD+HIIT **(F)** are displayed. In HFD animals, both exercise programs showed a trend to induce a reduction of this protein. A negative control without primary antibody is shown in **(G)**. The size bars indicate 100 μm and images are at 10× magnification. Quantification of positive staining is presented as means ± SEM and the number of animals in each group is 3. ^#^*p* = 0.07 and 0.08 vs. HFD untrained by Two-way ANOVA with Tukey’s and Sidak’s *post hoc* tests.

To assess the impact of HFD and exercise on the subcutaneous adipose tissue, we measured the mRNA levels of key structural and metabolic genes. As expected, collagen VI mRNA levels were higher HFD untrained mice, however, only after HIIT were these changes not observed (Figure 6A). To corroborate this finding, collagen VI protein levels were assessed by immunohistochemistry, finding that END and HIIT tended to exhibit lower levels of this protein in high-fat fed mice, (*p* = 0.08 and 0.07, respectively; Figure 7). Monocyte chemotactic protein-1 (Mcp-1) mRNA levels were higher in unexercised HFD mice and this difference was not seen after both exercise programs (Figure 6B). Glut1 mRNA levels were higher in CHOW-fed animals after END (Figure 6C), whereas no dietary or exercise effects were seen in Glut4 mRNA (Figure 6D). Higher transcriptional levels of adiponectin were observed in HFD mice and this was further increased by END, a change that was not seen in HIIT exercised animals (Figure 6E).

**FIGURE 8:**
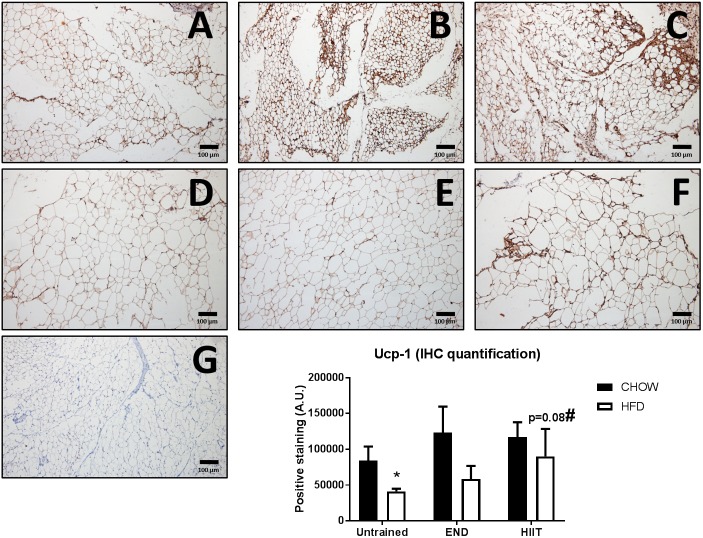
Immunohistochemistry and quantification of subcutaneous adipose tissue uncoupling protein 1 (Ucp-1). Representative cross-sections of subcutaneous adipose tissue with uncoupling protein 1 immunohistochemistry determined by immunoperoxidase stain, and nuclear counter-staining with haematoxylin. CHOW untrained **(A)**, CHOW+END **(B)**, CHOW+HIIT **(C)**, HFD untrained **(D)**, HFD+END **(E)**, and HFD+HIIT **(F)** are displayed. HFD diet induces a decrease of positive staining, whereas HIIT normalize this change. A negative control without primary antibody is shown in **(G)**. The size bars indicate 100 μm and images are at 10× magnification. Quantification of positive staining is presented as means ± SEM and the number of animals in each group is 3. ^∗^*p* < 0.05 vs. CHOW untrained; ^#^*p* = 0.08 vs. HFD untrained by Two-way ANOVA with Tukey’s and Sidak’s *post hoc* tests.

In terms of mitochondrial markers, HFD exhibit higher levels of mRNA levels of PR domain containing 16 (Prdm16; Figure 6F), Pgc-1 alpha (Figure 6G), and Mitochondrial transcription factor A (Tfam; Figure 6H), changes that were not seen after HIIT. No dietary or exercise effects were seen in Nuclear respiratory factor 1 (Nrf1) mRNA levels (Figure 6I). In contrast to the previously described genes, HIIT showed higher Ucp-1 mRNA levels in HFD animals (Figure 6J). To validate this finding, we measured the protein content of UCP-1 using immunohistochemistry, where we found that HFD untrained animals had less UCP-1 (*p* < 0.05), and only in HIIT exercise did this parameter tend to be higher (*p* = 0.08; Figure 8).

### Effects of Diet and Exercise on Liver

**FIGURE 9:**
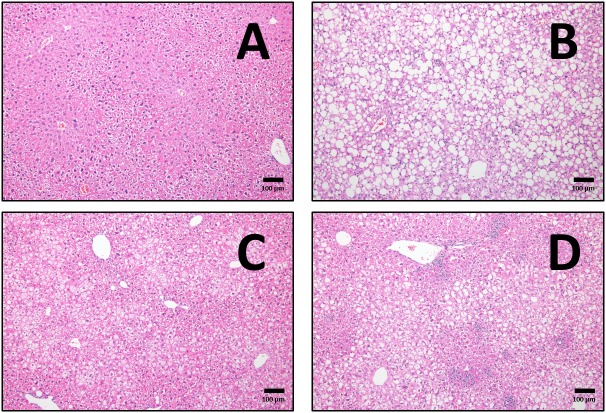
Representative cross-sections of liver, with haematoxylin and eosin staining at 10× magnifications for CHOW untrained **(A)**, high-fat fed untrained **(B)**, high-fat fed after endurance training (HFD+END; **C**), and high-fat fed after high-intensity interval training (HFD+HIIT; **D**). The effects of high-fat diet are clearly shown in **(B)** with the presence of lipid droplets, whereas both exercise programs induce a reduction in this finding. Since no major differences were seen in CHOW mice after exercise training, those images are not shown. The size bars indicate 100 μm and images are at 10× magnification.

**FIGURE 10:**
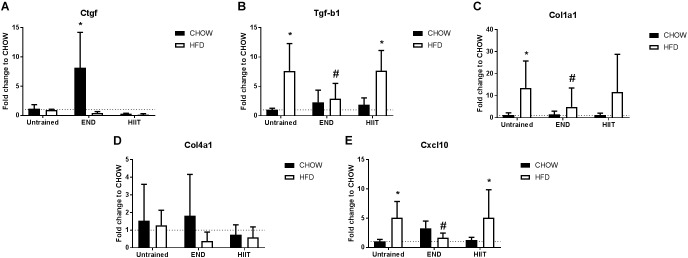
Liver mRNA levels of extracellular matrix and inflammation-related genes. Connective tissue growth factor/CCN2 (Ctgf; **A**), transforming growth factor beta (Tgf-b1; **B**), collagen 1 alpha 1 (col1a1; **C**), collagen 4 alpha 1 (col4a1, **D**), and interferon gamma-induced protein 10 (Cxcl10; **E**). Data are presented as means ± SD and the number of animals in each group is 2–9. ^∗^*p* < 0.05 vs. CHOW untrained; ^#^*p* < 0.05 vs. HFD untrained by Two-way ANOVA with Tukey’s and Sidak’s *post hoc* tests.

After 20 weeks of HFD, liver steatosis was clearly evident in the untrained mice, a phenomenon that was visibly different after both exercise regimes (Figure 9A–D). To study the effect of these interventions on tissue remodeling in fatty liver, we measured the mRNA and protein levels of key fibrotic and inflammatory markers as previously described (Lo et al., 2011; Yang et al., 2014). From a transcriptional point of view, HFD mice had higher levels of liver mRNA of TGF-β1 and collagen I (Col1a1), differences that were not seen after END (Figure 10B,C). In CHOW animals, END showed higher levels of Ctgf, also known as CCN2 (Figure 10A), whereas no dietary or exercise effects were seen in collagen IV (Col4a1; Figure 10D). HFD mice showed higher pro-inflammatory interferon gamma-induced protein 10 (Cxcl10) mRNA, difference that was milder in HFD+END animals (Figure 10E).

**FIGURE 11:**
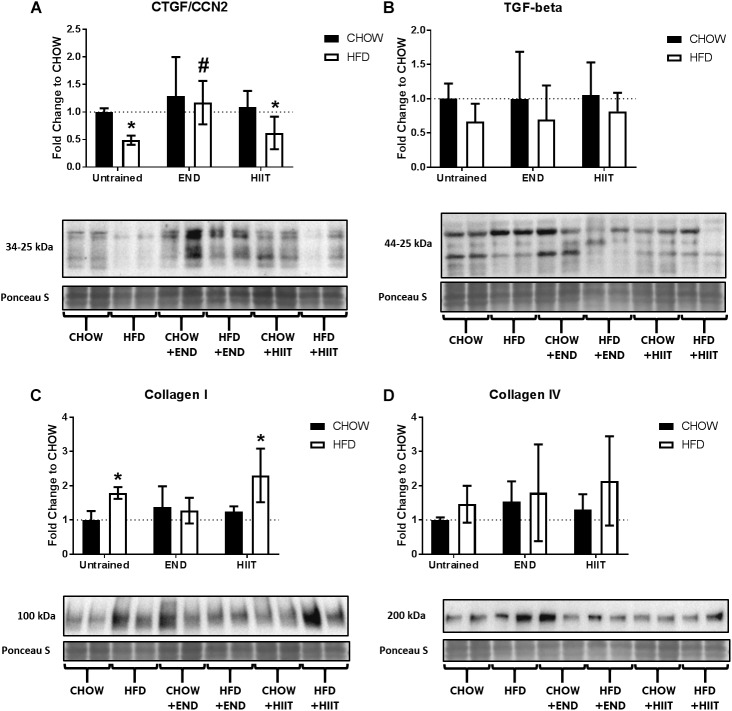
Liver collagens and growth factors. Connective tissue growth factor/CCN2 (CTGF/CCN2; **A**), transforming growth factor beta (TGF-β; **B**), collagen I **(C)**, collagen IV **(D)**, and their representative blots below its respective graph with its membrane stained with Ponceau S used as loading control. Data are presented as means ± SD and the number of animals in each group is 5–6. ^∗^*p* < 0.05 vs. CHOW untrained; ^#^*p* < 0.05 vs. HFD untrained by Two-way ANOVA with Tukey’s and Sidak’s *post hoc* tests.

In terms of protein content, HFD mice had lower CTGF/CCN2 protein, difference not present after END (Figure 11A and Supplementary Figure S7), whereas TGF-β did not show major differences (Figure 11B and Supplementary Figure S8). For liver collagens, HFD mice showed higher levels of collagen I protein, difference not seen after END (Figure 11C and Supplementary Figure S9), however, no dietary or exercise effects were seen on liver collagen IV protein (Figure 11D and Supplementary Figure S10).

## Discussion

To our knowledge, this is the first study that compares the metabolic effects of two distance and average intensity equivalent exercise programs on high-fat fed mice, targeting three insulin-sensitive tissues. Here, we found that 10 weeks of END and HIIT induced different metabolic benefits in this obese mouse model, which were dependent on the tissue analyzed. HIIT tended to favor improvements in skeletal muscle and subcutaneous white adipose tissue, whereas END was more efficient in treating the liver extracellular matrix (ECM) changes mediated by HFD. Interestingly, all the metabolic benefits described were independent of change in body weight or composition. This absence of changes was expected, considering that exercise programs without parallel dietary interventions does not affect body weight in a great extent (Winn et al., 2018). Considering that the animals had access to food *ad libitum*, compensatory adaptations in terms of food intake could be in place (Pomerleau et al., 2004), hypothesis that cannot be explored giving the limitation of this study associated with the absence of daily food intake records. To address this problem, “pair feeding” strategies are suggested in future experiments. Surprisingly, plasma insulin was unchanged by exercise in HFD groups, where even a significant increase in this outcome in HFD+END mice were seen. This could be explained by the fact that samples for this assay were collected before tissue harvest, therefore no fasting periods were considered and this random factor could have influenced this measurement. Interestingly, FGF-21 was similarly decreased by both exercise programs in HFD groups, as a change that could indicate that exercise induces a systemic sensitization to the action of this molecule, considering that obesity it has been reported as an FGF-21 resistant state (Fisher et al., 2010).

From a dietary perspective and similar to other reports, the expected differences in muscle total triglycerides after HFD were not altered by either END or HIIT (Jordy et al., 2015). This could be related to previous studies describing exercise-related changes in certain lipid species (Lee et al., 2016). Therefore, more sensitive methods (e.g., lipidomics) are required to assess the effect of different exercise prescriptions on the skeletal muscle lipid profile.

The metabolic benefits of exercise on the skeletal muscle are related in part, to its glucose uptake capacity and mitochondrial function. In this context, no changes in total GLUT4 protein nor in its localization by immunohistochemistry were seen throughout the groups, which can be expected given that samples were collected at least 72 h after the last bout of exercise. To explore the chronic effects of both exercise programs, our mouse tissue was deliberately harvested at a time well after the last exercise session. This enabled the more sustained changes in metabolic outcomes to be examined. In contrast to our data, studies in humans (Little et al., 1985) and in mice [albeit in for example db/db mice (Chavanelle et al., 2017)] demonstrating increases in skeletal muscle GLUT4 protein by HIIT, involved tissue sampling within 48 h after the last exercise session, at a time when effects of the last exercise session on GLUT4 protein may well be observed. Consistent with our data, previous studies have reported that exercise-related changes in muscle GLUT4 are no longer apparent 40 h after the last training session, given the short half-life of this protein and that acute effects of exercise on GLUT4 are transient (Host et al., 1998; Lehnen et al., 2012). We did observe exercise-related increases in the Phospho-AMPK^Thr172^/AMPK ratio in skeletal muscle, where particularly in CHOW-fed mice only, HIIT induced a significant increase. This finding suggests an exercise-intensity related activation of this pathway, as previously described in humans (Schwalm et al., 2015). However, in HFD mice exercise increased Phospho-AMPK^Thr172^/AMPK ratios irrespective of intensity, suggesting that in the context of chronic HFD intake, skeletal muscles respond similarly to exercise programs at different intensities regarding the activation of this pathway. The mechanisms behind this differing behavior should be investigated in future studies.

In terms of mitochondrial function, the role of muscle adiponectin was of particular interest in our study. Described as an insulin-sensitizer, anti-inflammatory and anti-oxidant (Kadowaki et al., 2006), adiponectin can also be found in skeletal muscles acting in an autocrine/paracrine manner (Jortay et al., 2012). Its actions are induced mainly by the activation of adiponectin receptor 1 (AdipoR1) (Jortay et al., 2012), and the consequent activation of sirtuin 1 (Sirt1) and PGC-1α (Iwabu et al., 2010). Notably, END exhibit higher transcriptional levels of muscle adiponectin that was inconsistent with the trend seen at the protein level. However, the expected lower levels of HMW isoform in HFD mice (Yang et al., 2006; Liu et al., 2009) was not seen after both exercise programs, although only after HIIT no significant differences in AdipoR1 were seen. Interestingly, no differences in the mRNA levels of adiponectin-related genes (Sirt1, Pgc-1alpha, and Ucp2) (Liu and Sweeney, 2014) were seen. This suggests that mechanistic studies are needed to examine whether the changes promoted by HIIT on AdipoR1 were functionally relevant.

The expansion of white adipose tissue is a hallmark of obesity, with inflammation, ECM accumulation, and mitochondrial dysfunction being the main features of this phenomenon (Spencer et al., 2010; Sun et al., 2013). To investigate whether END or HIIT may affect these outcomes, the mRNA level of Mcp-1 and collagen VI were measured. Intriguingly, the expected higher levels of Mcp-1 (Gleeson et al., 2011) were not seen after both exercise regimens. Moreover, END and HIIT were effective in keeping lower in collagen VI levels in this tissue. These results collectively suggest that the benefits of exercise on adipose tissue inflammation and ECM accumulation are independent of exercise intensity. Further experiments are required to investigate the underlying mechanisms. Intriguingly in this study, despite the important role of white adipose tissue (together with skeletal muscle) in Glut1 and Glut4 receptor-mediated glucose uptake (Rosen and Spiegelman, 2006), only higher mRNA levels of the former were seen in lean mice after END. However, further experiments should aim to elucidate the protein changes of these receptors after acute, acute over chronic, and chronic adaptations derived from END and HIIT regimes, in order to have a deeper understanding of the effects of exercise on glucose uptake in white adipose tissue during obesity.

One commonly described adipokine is adiponectin (Lee and Kwak, 2014). Interestingly, exercise did not modify the plasma adiponectin content in any of its isoforms (LMW or HMW), which conflicts with some previous studies that associate hypoadiponectinaemia with obesity (Weyer et al., 2001; Di Chiara et al., 2012; Prakash et al., 2013). Nevertheless, a large study conducted by Kuo and Halpern (2011) failed to show any association between plasma adiponectin levels and body mass index in almost 5000 subjects, even when both overweight and obese people were included. This may suggest that obesity *per se* is not the main cause of hypoadiponectinaemia.

White adipose tissue “browning” has been associated with increased mitochondrial function (Rodriguez et al., 2017), benefits of exercise (Ringholm et al., 2013; Stanford et al., 2015; Wang et al., 2017) and aerobic performance in mice (Brenmoehl et al., 2017). In this regard, only after HIIT higher UCP1 mRNA and protein levels in the subcutaneous adipose tissue were detected in HFD mice, similar to what others have similarly described (Wang et al., 2017). Interestingly, and as showed by others (Mossenbock et al., 2014) these changes in subcutaneous adipose tissue seem to be independent of systemic insulin levels, given that in our study exercise did not reverse hyperinsulinemia. There is a possibility that the well-known higher adrenergic input associated with high-intensity exercise (Weltman et al., 1994) favored the browning process compared to a moderate intensity protocol. However, mechanistic studies are needed to elucidate the differential mediators between END and HIIT that makes the latter more efficient at inducing browning of white adipose tissue in a HFD context.

Another prevalent complication of obesity is non-alcoholic fatty liver disease (NAFLD) (Fabbrini et al., 2010) which has been associated with ectopic fat deposition and the development of insulin-resistance, inflammation, and fibrosis (Moore, 2010). In this context, the level liver fat in HFD mice was clearly lower after exercise. Interestingly, others have seen similar findings when comparing END and HIIT after 12 weeks of END and HIIT in OLEFT rats (Linden et al., 2015). Cho et al. (2015) described a greater metabolic protection in the liver from HIIT compared to END in high-fat fed C57BL/6 mice after 8 weeks of training. However, the intensity at which mice ran in the END protocol in that study was very low (10 m/min, compared to 15 m/min in this study). When the protocols are isocaloric, the liver metabolic differences between END and HIIT tend to disappear. This was seen by Winn et al. (2018) who in a study of overweight/obese people observed that after 4 weeks of energy-matched END and HIIT, both exercise programs conferred similar improvements in intrahepatic fat and lipid peroxidation levels without any changes in body weight or body composition. This suggests that when exercise programs are isocaloric, they are similarly able to reverse the liver fat deposition and tissue damage derived from obesity.

Regarding the liver extracellular matrix, the higher levels of TGF-β (mRNA) and collagen I (mRNA and protein) with HFD were not appreciated only after END. Similar to the mRNA levels of Cxcl10, which has recently been described as a relevant inflammatory mediator in NAFLD (Zhang et al., 2014). Considering these findings, the HFD-mediated decrease of CTGF/CCN2 could be seen as counterintuitive in this pro-fibrotic and inflammatory profile. However, it has been previously described that this growth factor is sensitive to the action of pro-inflammatory cytokines (Abraham et al., 2000; Laug et al., 2012), which reduce its expression. This further supports the presence of inflammation in livers of HFD untrained animals. Altogether, these data suggest that END is more efficient at reducing HFD-derived liver inflammation, protecting the ECM from abnormal remodeling. Nevertheless, more studies regarding the differential metabolic pathways that regulate these responses are urgently needed.

This study highlights that exercise regimens equivalent in terms of distance covered and time per session as well as in average exercise intensity, but at different maximal intensities, appear to elicit differential metabolic effects in insulin-sensitive tissues. The exact mechanisms behind this are, to date, unknown. However, the intensity-dependence of the insulin sensitizing effect of exercise (Wojtaszewski et al., 2000; Parker et al., 2017), and the higher systemic adrenergic inputs described after high-intensity exercise (Weltman et al., 1994), might preferentially induce white adipose browning after HIIT over END. Interestingly, exercise benefits on liver metabolism seem to be not directly associated with higher exercise intensity, given the specific benefits of END on this tissue. This could suggest that the source of fuel utilization (e.g., carbohydrates vs. lipids) might be more relevant, particularly in a HFD context, where END is known to preferentially induce fat oxidation over glucose utilization (van Loon et al., 2001), thus at least partially countering the high levels of fat intake through diet.

**FIGURE 12:**
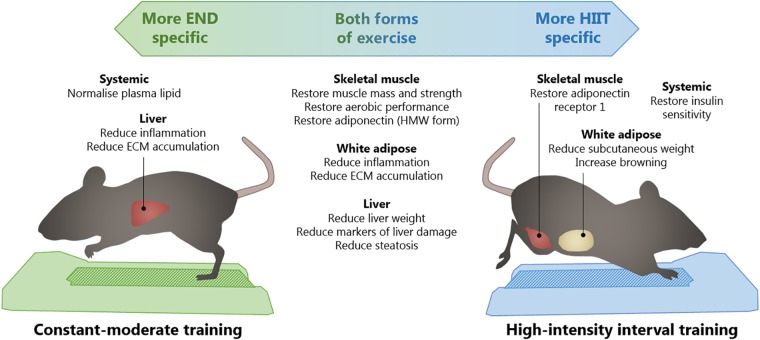
Summary of proposed clinical implications of different exercise programs in the normalization of outcomes of high-fat diet. Across the two running subjects, common benefits of constant-moderate endurance (END) and high-intensity interval training in skeletal muscle, subcutaneous adipose tissue, and liver, are listed. On the left, specific putative benefits from END are described and, on the right, benefits specific to HIIT are listed. High molecular weight (HMW); extracellular matrix (ECM).

The clinical implications of this study reside in that, even when exercise programs are matched to expend a similar amount of energy, exercise could be tailored to impact specific patient sub-phenotypes and tissues depending on the dysfunction present. This is particularly interesting, knowing that the obese phenotype is often variable between individuals in its metabolic profile (Chen et al., 2015). In this context, clinical studies regarding HIIT have been highly focused on its time-efficiency and enjoyability (Bartlett et al., 2011). However, while both types of exercise can deliver common metabolic benefits in an environment of high fat feeding as shown in the current preclinical research, HIIT may be relatively more beneficial in clinical states where whole body insulin resistance dominates, whereas END may well be of relatively greater benefit in protecting against development of more advanced inflammatory and fibrotic NAFLD known as non-alcoholic steatohepatitis. Future research in humans will be important to determine whether the relative advantages highlighted in the Schematic in Figure 12 seen in the current rodent research, are borne-out in clinical settings of excess caloric intake in obesity, and effects of tailored exercise regimens.

## Conclusion

In summary, our data suggests that when END and HIIT are designed to be equivalent in terms of distance covered, timing per session, and average intensities, similar effects can be seen in the metabolic function of skeletal muscle, white adipose tissue, and liver. Interestingly, these changes are independent of variations in body weight or body composition. Nevertheless, HIIT seems to induce further benefits on the level of adiponectin receptors in skeletal muscle and the browning of white adipose tissue, whereas END is more effective in protecting the liver ECM against disturbances induced by HFD (Figure 12). This indicates that different exercise prescriptions may provide favorable outcomes to different insulin sensitive tissues, a finding that with time will likely be considered when physical exercise is prescribed to manage metabolic disturbances associated with obesity. Future studies that explore the mechanisms behind these differential benefits would further clarify the differential outcomes described in this study.

## Ethics Statement

This study was approved by the University of Sydney Animal Ethics Committee (Protocol #2015/816). The experiments described herein were carried out according to the guidelines laid down by the New South Wales Animal Research Act and the 8th Edition of the Australian code for the care and use of animals for scientific purposes.

## Author Contributions

SM-H, BM, PW, CT, SM, and ST conceptualized and designed the experiments. SM-H, LB, LO-A, and BM executed the experiments. SM-H and LB analyzed the data and prepared the figures. SM-H, LB, PW, CT, SM, and ST interpreted the results. SM-H, LB, CT, and ST drafted the manuscript. SM-H, LB, LO-A, BM, PW, CT, SM, and ST edited and revised the manuscript and approved the final version of manuscript.

## Conflict of Interest Statement

The authors declare that the research was conducted in the absence of any commercial or financial relationships that could be construed as a potential conflict of interest.
