# Supplementary material for: Constant-Moderate and High-Intensity Interval Training Have Differential Benefits on Insulin Sensitive Tissues in High-Fat Fed Mice
Source: Front Physiol. 2019 Apr 25;10:459. doi: 10.3389/fphys.2019.00459 (PMC6494961; doi:10.3389/fphys.2019.00459)
Supplement: Supplementary file 1 [file Data_Sheet_1.PDF]

Supplementary Information

Constant-moderate and high-intensity interval training have differential benefits on insulin sensitive tissues in high-fat fed mice.

Sergio F Martinez-Huenchullan[1,2], Linda A Ban[1], Luisa F Olaya-Agudo[1], Babu Raja Maharjan[1], Paul F Williams[1], Charmaine S Tam[4], Susan V Mclennan[1,5,6], Stephen M Twigg[1,6] *

Supplementary table 1. Primers used for rt-qPCR

Gene	Forward	Reverse
Glut4	5’-CCAGTATGTTGCGGATGCTAT-3’	5’-ATCTGGTCAAACGTCCGGC-3’
Hexokinase 1	5’-TCGGAGGAACGAATTTCCGAGT-3’	5’-ACAATGTGATCAAACAGCTCATCC-3’
Hexokinase 2	5’-TCCAGACGGTACAGAGAAAGGA-3’	5’-TCTCTACGCCCCTTCGCTTG-3’
Mcp-1	5’-CACTCACCTGCTGCTACTCA-3’	5’-GCTTGGTGACAAAAACTACAG-3’
Adiponectin	5’-CGACACCAAAAGGGCTCAGG-3’	5’-ACGTCATCTTCGGCATGACT-3’
AdipoR1	5’-GCAGACAAGAGCAGGAGTGT-3’	5’-TTGACAAAGCCCTCAGCGAT-3’
Sirtuin 1	5’-AGCGGCTTGAGGGTAATCAA-3’	5’-GAGTATACCTCAGCACCGTGG-3’
Pgc-1α	5'-CTGCGGGATGATGGAGACAG-3'	5'-TCGTTCGACCTGCGTAAAGT-3'
Ucp2	5’-GGCCTCTGGAAAGGGACTTCT-3’	5’-TTGGCTTTCAGGAGAGTATCTTT-3’
Tnf	5'-GACCCTCACACTCACAAACCA-3'	5'-ACAAGGTACAACCCATCGGC-3'
Col6a1	5’-GATGAGGGTGAAGTGGGAGA-3’	5’-CAGCACGAAGAGGATGTCAA-3’
Ucp-1	5'-CATGGGATCAAACCCCGCTA-3'	5'-ATTAGGGGTCGTCCCTTTCC-3'
Glut1	5’-AGTATGTGGAGCAACTGTGCG-3’	5’-CCGGAAGCGATCTCATCGAA-3’
Prdm16	5’-TGACCATACCCGGAGGCATA-3'	5'-CTGACGAGGGTCCTGTGATG-3'
Tfam	5’-GAGCGTGCTAAAAGCACTGG-3’	5’-CAGACAAGACTGATAGACGAGGG-3’
Nrf1	5’-ACAAGGTGGGGGACAGATAGT-3’	5’-ATCTGGACCAGGCCATTAGC-3’

Rpl7L1	5’-ACGGTGGAGCCTTATGTGAC-3’	5’-TCCGTCAGAGGGACTGTCTT-3’
NoNo	5'-TGCTCCTGTGCCACCTGGTACTC-3'	5'-CCGGAGCTGGACGGTTGAATGC-3'
	Taqman format	
Ctgf	Mm01192932_g1	
Tgf-beta	Mm01178820_m1	
Col1a1	Mm00801666_g1	
Col4a1	Mm01210125_m1	
Cxcl10	Mm00445235_m1	
AldoB	Mm00523293_m1	

Supplementary table 2. List of antibodies and working dilutions.

Target protein	Brand	Catalog number	Dilution
Adiponectin	GeneTex	GTX23455	1:2000 (WB)<br>1:500 (IHC)
AdipoR1	GeneTex	GTX32425	1:2000 (WB)
Phospho-AMPK^{Thr172}	Cell Signaling	2535S	1:1000 (WB)
AMPKα	Cell Signaling	2532S	1:1000 (WB)
Collagen I	Abcam	Ab6308	1:2500 (WB)
Collagen IV	Abcam	Ab6586	1:2500 (WB)
Collagen VI	Abcam	Ab6588	1:500 (IHC)
CTGF/CCN2	GeneTex	GTX124232	1:2000 (WB)
TGFβ1	Abcam	Ab92486	1:2000 (WB)
GLUT4	Abcam	Ab654	1:2500 (WB)<br>1:400 (IHC)
UCP1	Abcam	ab10983	1:500 (IHC)

WB: dilution used in western immunoblot; IHC: dilution used in immunohistochemistry

Plasma adiponectin

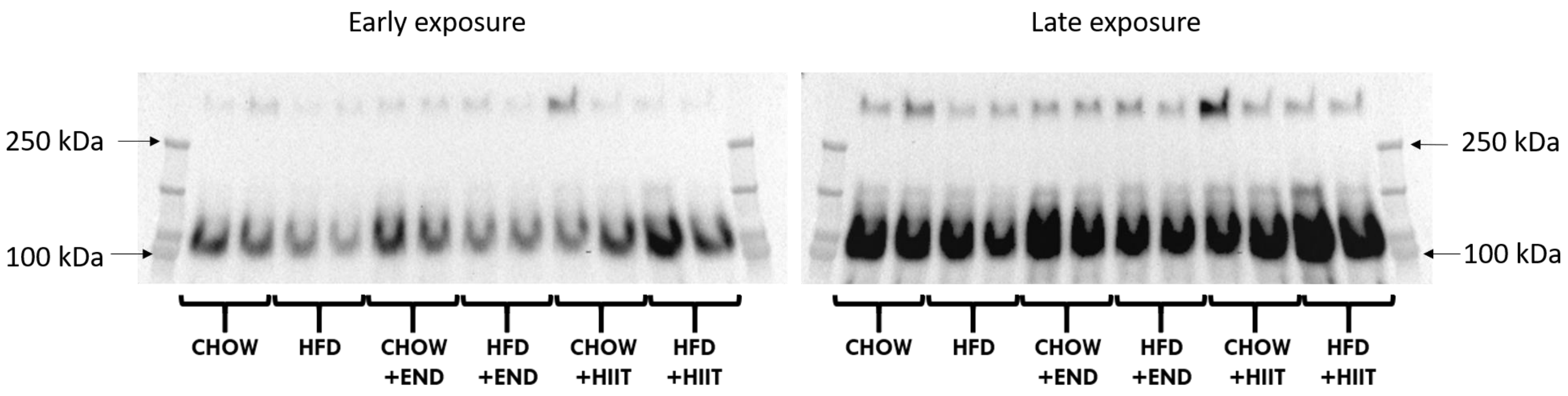

Supplementary figure 1. Representative blots of plasma adiponectin. Blots from Low-molecular (LMW) and High-molecular weight (HMW) adiponectin isoform were extracted from the same membrane with different exposure times.

Muscle adiponectin

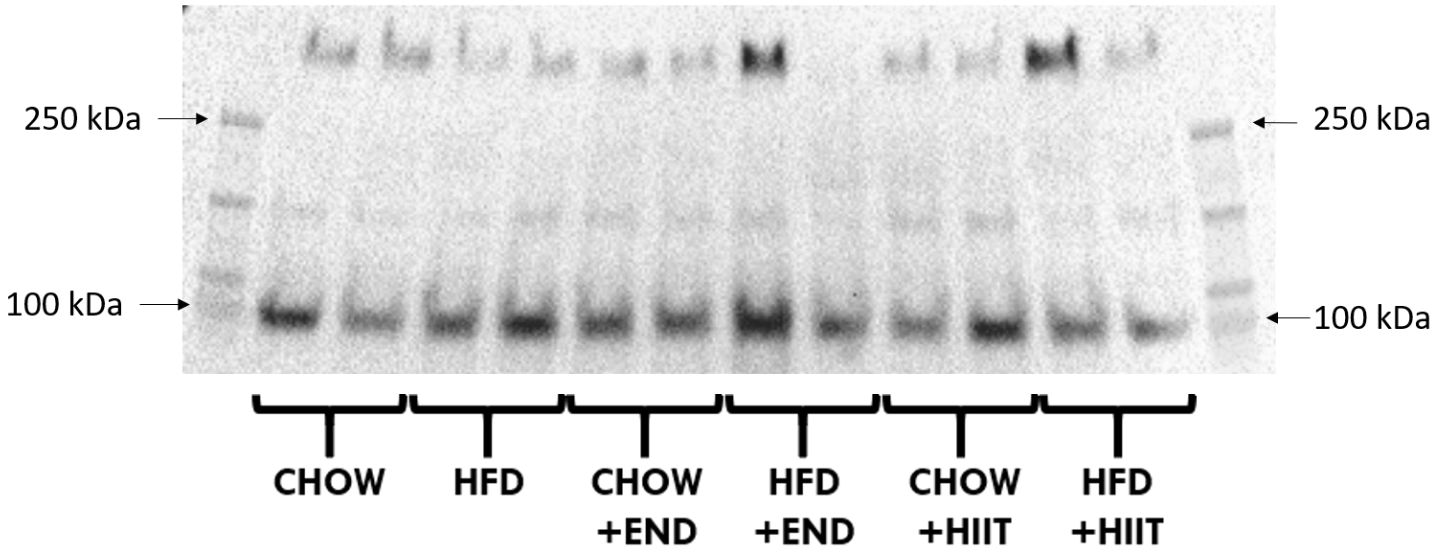

Supplementary figure 2. Representative blots of muscle adiponectin. Blots from Low-molecular (LMW) and High-molecular weight (HMW) adiponectin isoform were extracted from the same membrane with the same exposure time.

Muscle AdipoR1

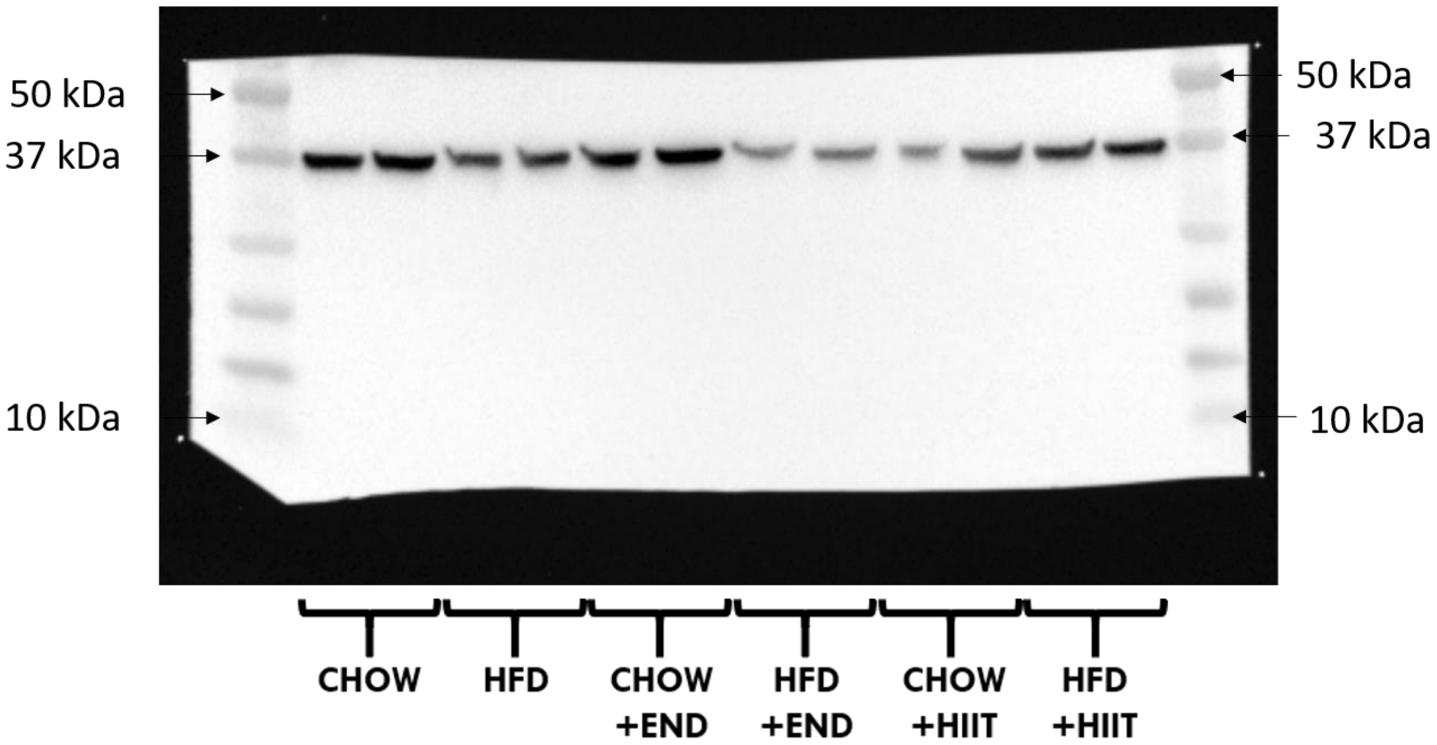

Supplementary figure 3. Representative blot of muscle AdipoR1.

Muscle GLUT4

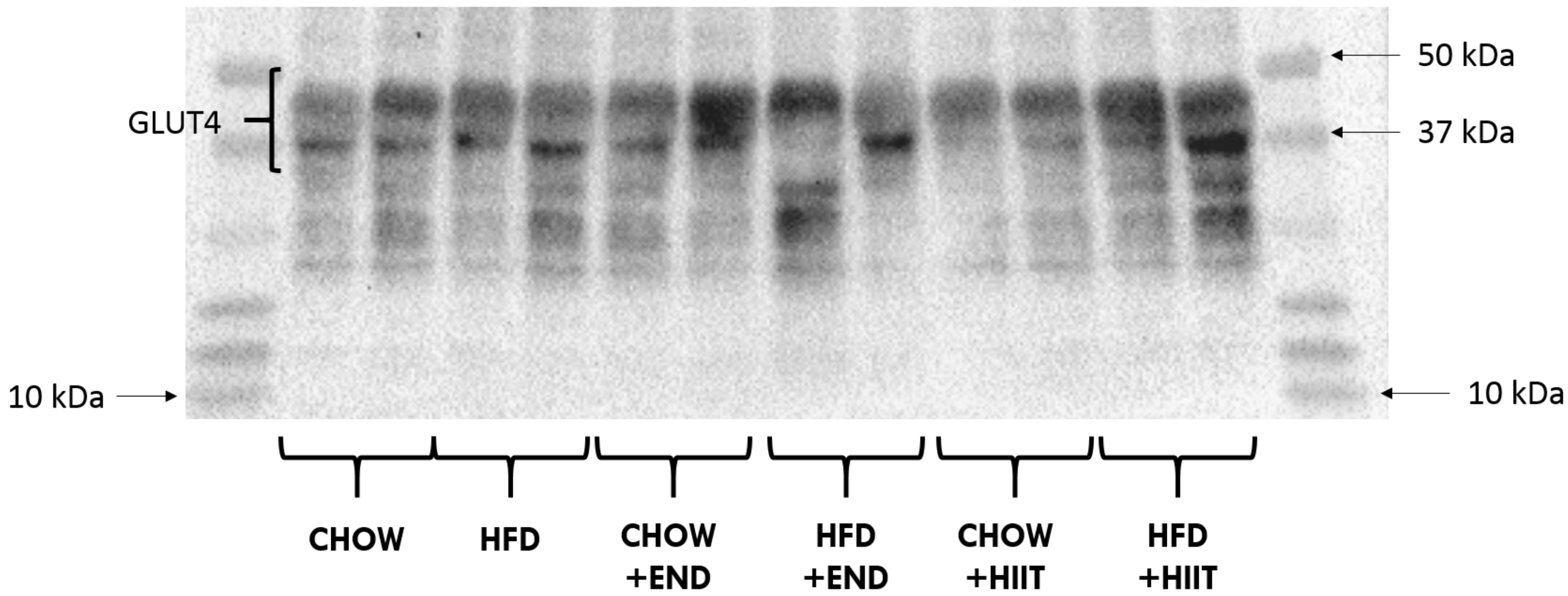

Supplementary figure 4. Representative blot of muscle GLUT4.

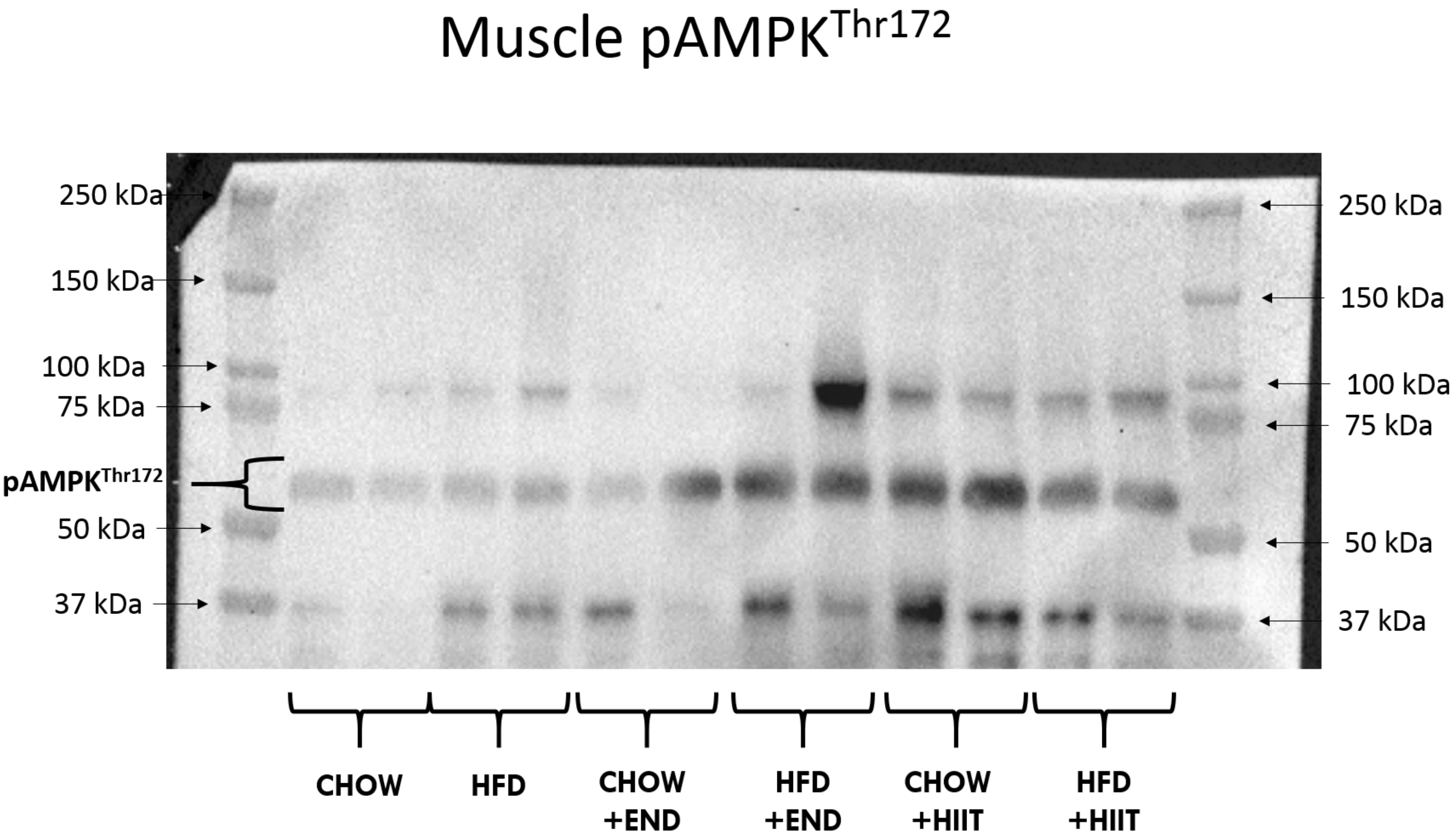

Supplementary figure 5. Representative blot of muscle Phospho-AMPK^{Thr172}.

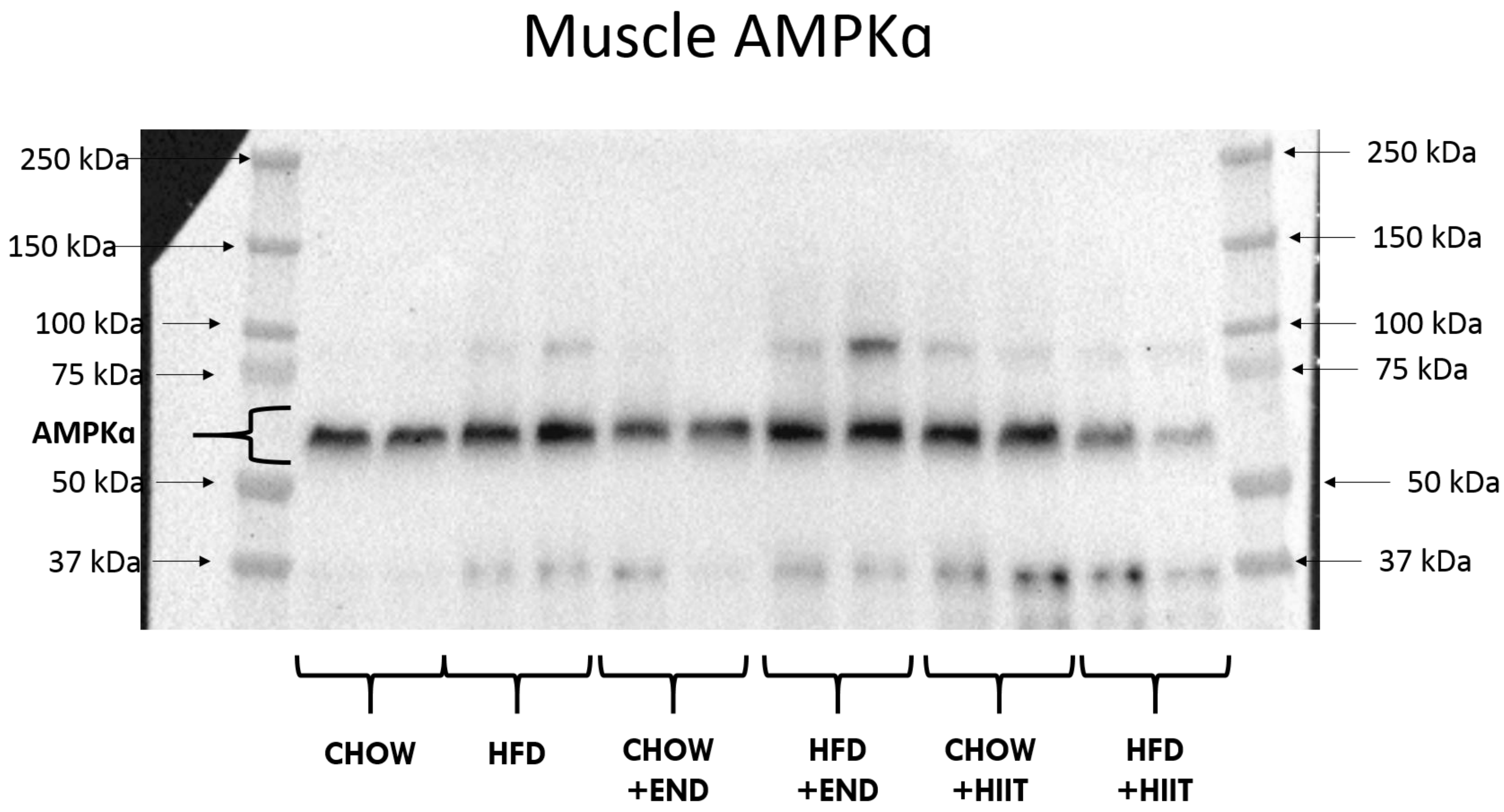

Supplementary figure 6. Representative blot of muscle AMPKα.

Liver CTGF/CCN2

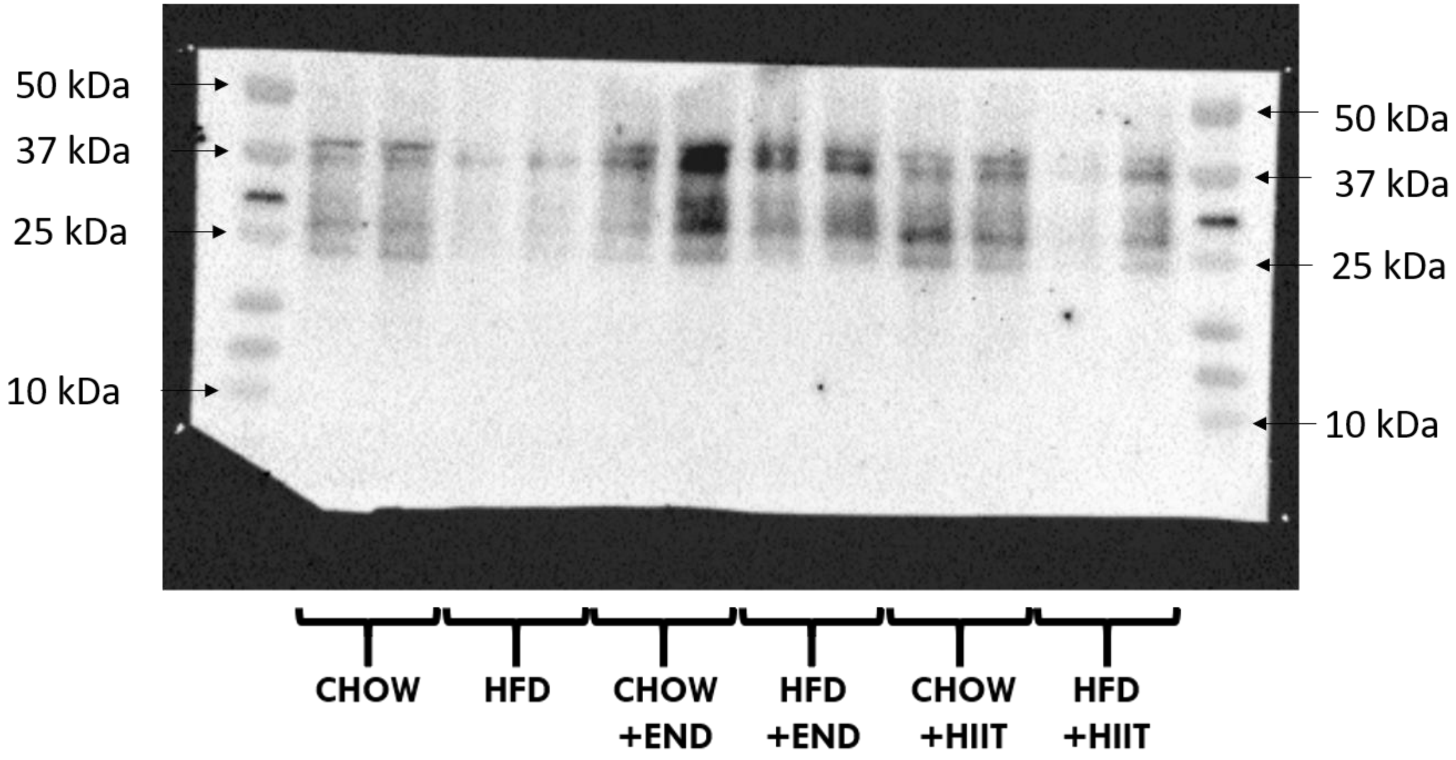

Supplementary figure 7. Representative blot of liver CTGF/CCN2.

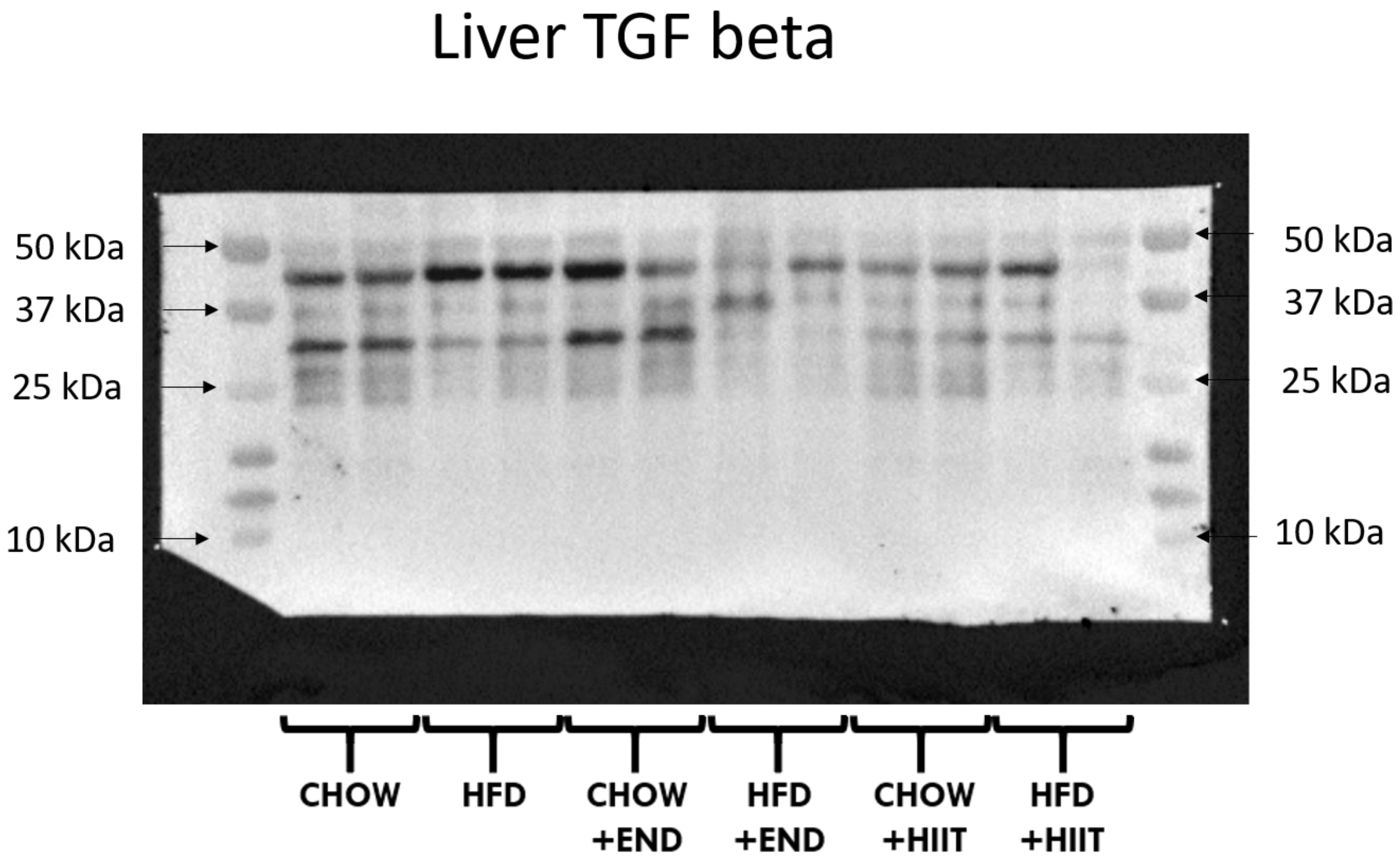

Supplementary figure 8. Representative blot of liver TGF beta.

Liver Collagen I

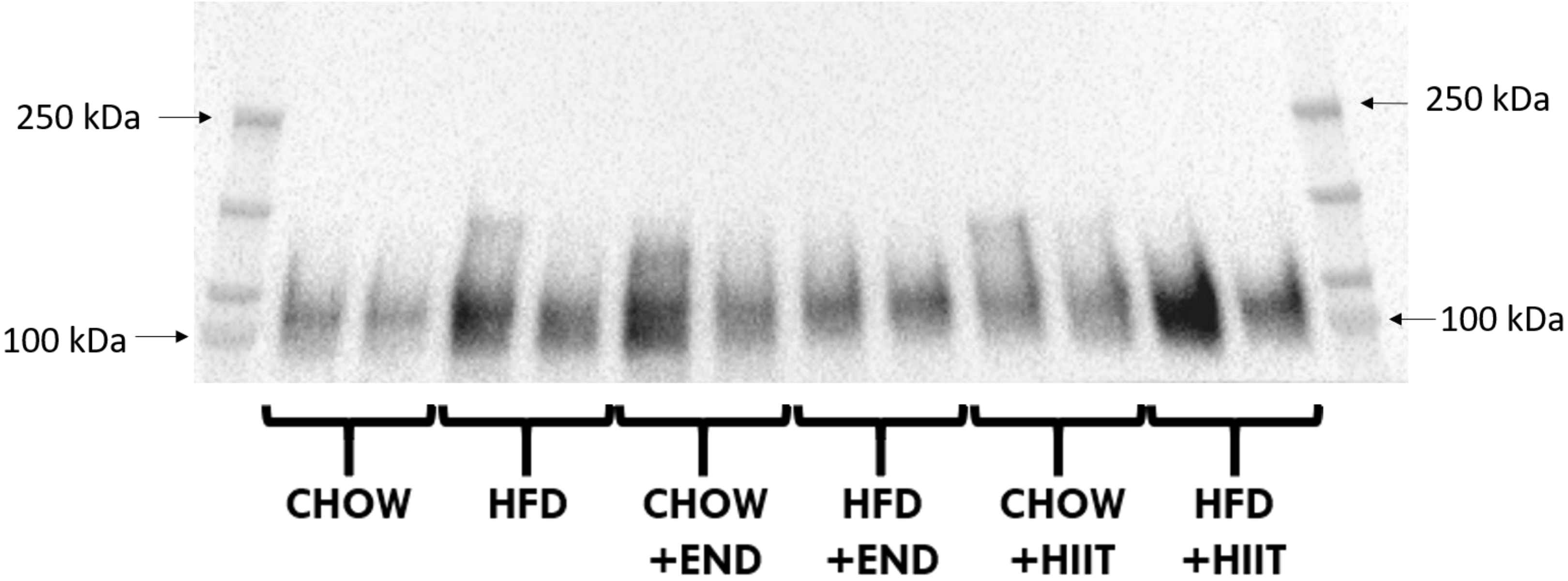

Supplementary figure 9. Representative blot of liver Collagen I.

Liver Collagen IV

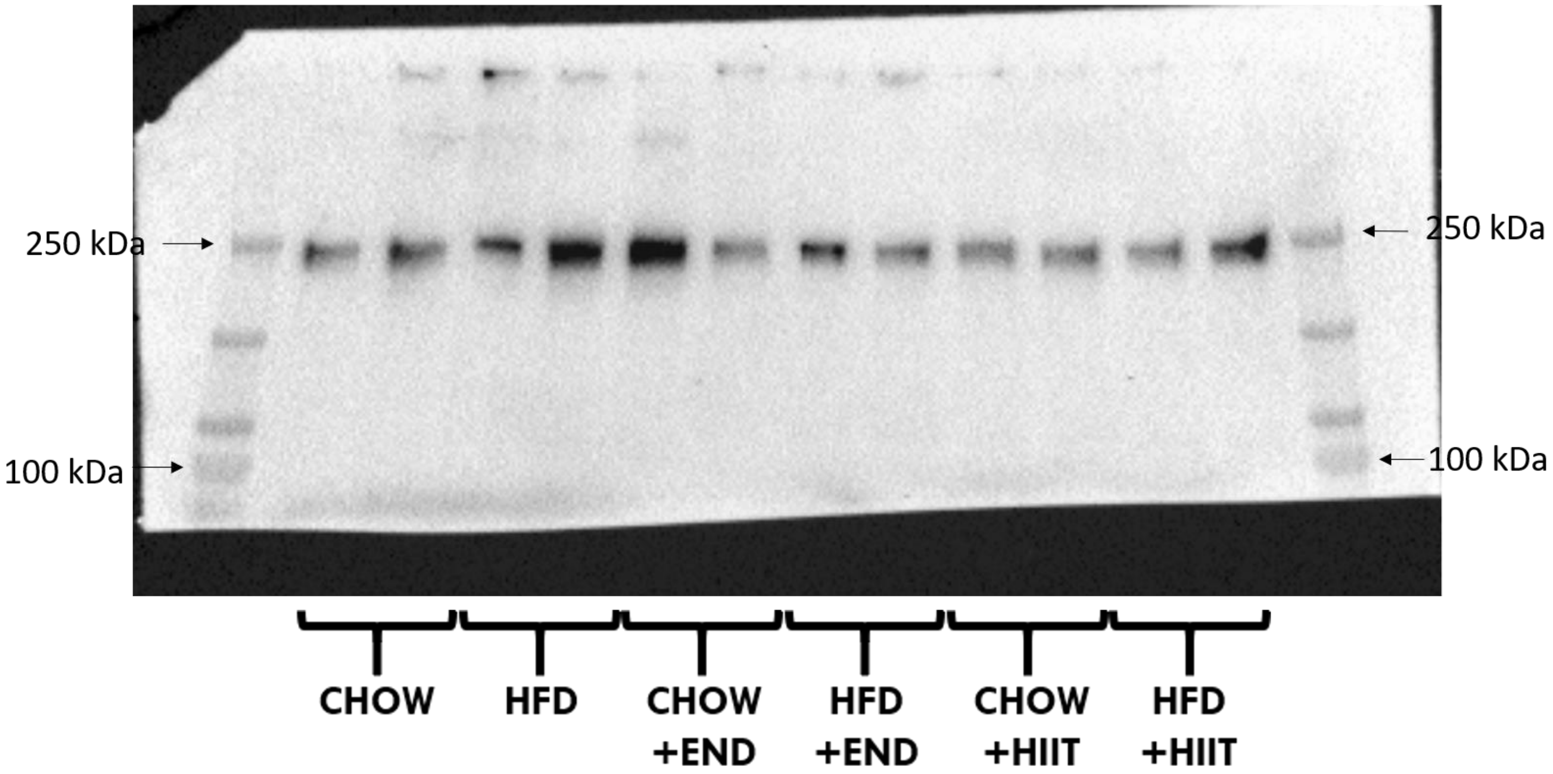

Supplementary figure 10. Representative blot of liver Collagen IV.
